# An Iterative Distortion Compensation Algorithm for Camera Calibration Based on Phase Target

**DOI:** 10.3390/s17061188

**Published:** 2017-05-23

**Authors:** Yongjia Xu, Feng Gao, Hongyu Ren, Zonghua Zhang, Xiangqian Jiang

**Affiliations:** 1 EPSRC Center, University of Huddersfield, Huddersfield HD1 3DH, UK; Yongjia.Xu@hud.ac.uk (Y.X.); Hongyu.Ren@hud.ac.uk (H.R.); x.jiang@hud.ac.uk (X.J.); 2 School of Mechanical Engineering, Hebei University of Technology, Tianjin 300130, China; zhzhang@hebut.edu.cn

**Keywords:** camera calibration, distortion compensation, phase measurement

## Abstract

Camera distortion is a critical factor affecting the accuracy of camera calibration. A conventional calibration approach cannot satisfy the requirement of a measurement system demanding high calibration accuracy due to the inaccurate distortion compensation. This paper presents a novel camera calibration method with an iterative distortion compensation algorithm. The initial parameters of the camera are calibrated by full-field camera pixels and the corresponding points on a phase target. An iterative algorithm is proposed to compensate for the distortion. A 2D fitting and interpolation method is also developed to enhance the accuracy of the phase target. Compared to the conventional calibration method, the proposed method does not rely on a distortion mathematical model, and is stable and effective in terms of complex distortion conditions. Both the simulation work and experimental results show that the proposed calibration method is more than 100% more accurate than the conventional calibration method.

## 1. Introduction

Camera calibration is the first and most essential step in optical 3D measurement such as stereo vision [1,2], fringe projection techniques [3,4,5] and deflectometry [6,7,8]. There are generally two aspects affecting the accuracy of camera calibration. One is the accuracy of the chosen camera model. The other is the location accuracy of the feature points in terms of the world coordinate system. Camera distortion is a critical factor affecting the accuracy of the camera model. The pinhole model is popularly adopted for the description of a general imaging process, which is a linear projection between a 3D point in the world and the corresponding 2D image in a camera. However the real imaging process is a nonlinear projection due to camera distortion. Camera lens distortion can be classified as radial distortion, eccentric distortion and thin prism distortion. The parameter-based approaches are popularly researched to compensate lens distortion [1,9,10]. Alvarez et al. [11] proposed a mathematical model to study the distortion variation of zoom lenses. Santana-Cedrés et al. [12] researched to estimate the distortion model by minimizing a line reprojection error. Conventional calibration approaches use infinite high order polynomials with distortion parameters to express the distortion caused from different sources [1,9,10]. Initial distortion parameters are estimated firstly, and then an optimization algorithm is applied to optimize the distortion parameters with other camera parameters based on the least squares method. Since the accuracy of the input plays an important role in the constringency of the iterative optimization, the accuracy of camera calibration is highly dependent on the accuracy of the initial distortion parameters. Moreover, the real distortion is the result of the cumulative effects of a complex lens system, the camera geometry error, and the imperfect shape of the image sensor; therefore a model with enough parameters is required to simulate any plausible distortion [13]. However, popular conventional calibration [1,10,11,12,13,14,15] has to only apply a distortion model with partial distortion parameters of radial distortion and eccentric distortion; experiments show that a more elaborative model would not help to improve accuracy but would also cause numerical instability [1]. Therefore, the applied distortion mathematical model of the conventional method cannot match the real distortion perfectly, which also restricts the calibration accuracy. In order to tackle this problem, non-parametric distortion compensation methods have been studied. Thirthala et al. [16] proposed an approach to recover radial distortion using multifocal tensors; however this approach is based on the assumption that the center of distortion is at the image center, which is generally not a safe assumption. Hartley et al. [17] proposed a parameter-free calibration approach that only considers the radial distortion. Ryusuke et al. [18] proposed a method to compensate lens distortion by using binary structured-light, however this approach cannot calibrate the internal parameter of a camera. Therefore, the investigation of a flexible, stable method is needed for calibrating the camera’s internal parameters, external parameters, and distortion.

Usually, a set of 2D or 3D control points or feature points with known world coordinates are used as input data for the calibration process [1,10]. Compared to 3D targets, 2D patterns such as checkerboards, squares and circles on a plane have been used frequently because they are easily manufactured and handled [1,19,20]. Recently, a 2D phase target has been studied as a substitute for traditional planar targets due to the advantage of its highly accurate feature detection, massive arbitrary provision of control points, and full automatic process [21,22]. Xue et al. [23] presented a method for camera calibration based on the orthogonal vanishing point calibration using concentric circles grating and wedge grating. Liu et al. [24] introduced a crossed-fringe pattern as the model plane for camera calibration. Ma et al. [25] proposed a feature extraction method by using fringe pattern groups as the calibration target. Moreover, a phase target can effectively calibrate the camera with a small depth of focus and long working distance because the obtained phase from sinusoidal fringe has little influence on out-of-focus images [26,27]. Schmalz et al. [18] made a comparison of camera calibration with a phase target and a classic planar target to show the superiority of the phase target.

In order to increase calibration accuracy, methods have been studied to decrease the location error of phase target. Schmalz et al. [21] proposed a fitting approach to smooth the phase value based on four neighboring pixels. Huang et al. [22] applied a windowed polynomial fitting technique to optimize the phase target and compared the result with linear interpolation, biquadratic fitting, and bicubic fitting. There were few details given for the principle and calculation process. A windowed bicubic fitting with a window size of 200 × 200 pixels was used in the experiment. However, the reasons why the window size was chosen and how much the window size affected the results were not provided. Though a remarkable reprojection accuracy was achieved, the feasibility of the window size needed to be discussed. The techniques mentioned above focus on the research of the calibration target design and the improvement of location accuracy of feature detection. However, no research has been done to improve the compensation accuracy of the camera distortion and to optimize the camera model with phase target.

This paper proposes a novel camera calibration method with an iterative distortion compensation algorithm. Full-field camera pixels and the corresponding points on a phase target are used to calculate calibration parameters. The deviation caused by distortion between the real pixel and the reprojection pixel based on a linear projection can be obtained. A corrected coordinate for the pixel can be obtained by compensating the deviation. By using the Levenberg–Marquardt algorithm, the calibration parameters are iteratively optimized by minimizing the difference between the corrected coordinate and the reprojection pixel. To enhance the location accuracy of the world coordinates, fitting techniques and the feasibility of the window size applied to the phase target have been presented to compensate for any phase error.

## 2. Principle and Methods

### 2.1. Phase Target

**Figure 1:**
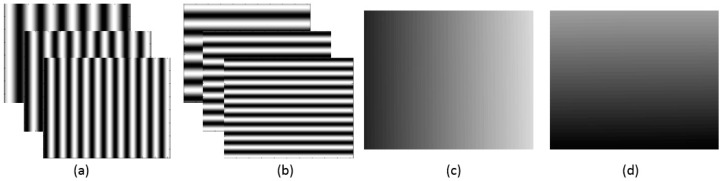
Phase target. (**a**) Vertical fringe patterns; (**b**) horizontal fringe patterns; (**c**) vertical phase map; (**d**) horizontal phase map.

A phase target is used to assist in the proposed calibration method for this paper (as shown in Figure 1). Apart from the advantages of the phase target as described in the introduction, the main reason for using a phase target is because it is a dense and continuous target and satisfies the requirement of the proposed distortion compensation algorithm. The proposed distortion compensation method is required to calculate the deviation between the real pixel and the reprojection pixel in terms of different calibration poses. When using the phase target, the camera pixel can find the corresponding world points in terms of different calibration poses based on the continuous phase maps. While a traditional 2D calibration board (such as the chessboard) is used, the proposed calibration method will fail because the same camera pixel cannot discover the corresponding world points in different calibration poses due to the limited and discrete feature points. Two groups of mutually perpendicular phase-shifting fringe patterns are displayed on a liquid crystal display (LCD) screen in sequence, and these displayed patterns are captured by a camera from different viewpoints. After applying the phase shifted method and the phase unwrapping method [28,29,30], two mutually perpendicular absolute phase maps are obtained, as shown in Figure 1. If the size of LCD pixel pitch p and the number $n_{p}$ of LCD pixels per fringe period are known, one camera pixel can uniquely locate its corresponding physical position $\left(x_{w},y_{w}\right)$ in the world coordinate system based on its phase value $\left(\varphi_{x},\varphi_{y}\right)$ according to Equations (1) and (2).

(1) $$x_{w}=(n_{p}\cdot p/2\pi )\cdot \varphi_{x}$$

(2) $$y_{w}=(n_{p}\cdot p/2\pi )\cdot \varphi_{y}$$

### 2.2. Calibration with Iterative Distortion Compensation Algorithm

**Figure 2:**
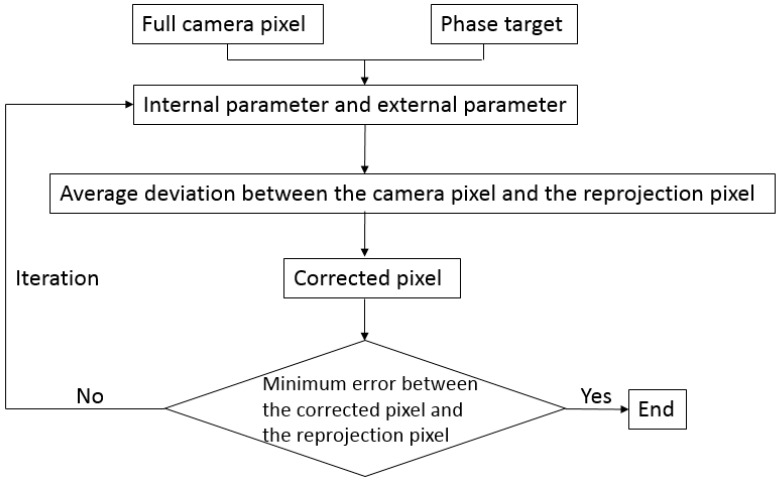
Calibration process of the proposed method.

**Figure 3:**
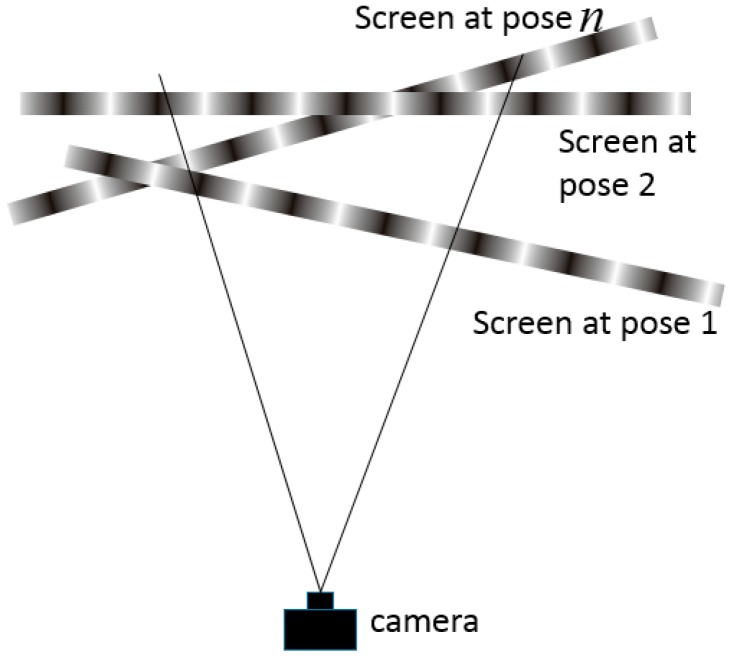
Calibration poses.

The proposed camera calibration process is illustrated in Figure 2. Two groups of orthogonal fringe patterns are displayed on a LCD screen in sequence. Images at arbitrary *n* LCD poses are captured during the whole calibration process, as shown in Figure 3. In terms of each LCD pose, *k* camera pixels uniformly distributed on a CCD (charge coupled device) plane are selected to calibrate the parameters. The selected pixels can be full pixels or sampled pixels with the same gap in order to enhance calculation speed. The corresponding world coordinates are obtained based on their phase values according to Equations (1) and (2). A linear projection *H* can be obtained according to Equation (3).

(3) $$s\left[\begin{array}{c} u\\ v\\ 1 \end{array}\right]=A\cdot \left[\begin{array}{cc} R & t \end{array}\right]\cdot \left[\begin{array}{c} x_{w}\\ y_{w}\\ z_{w}\\ 1 \end{array}\right]=H\left[\begin{array}{c} x_{w}\\ y_{w}\\ 1 \end{array}\right]\;\mathrm{with}\;A=\left[\begin{array}{ccc} \alpha & 0 & u_{0}\\ 0 & \beta & v_{0}\\ 0 & 0 & 1 \end{array}\right]$$

where (u,v) is the camera pixel coordinate and $\left(x_{w},y_{w}\right)$ is the corresponding world coordinate. A is the internal parameter of a camera, $\left(u_{0},v_{0}\right)$ are the coordinates of the principal point, α and β are the scale factors in image u and v axes. $\left[\begin{array}{cc} R & t \end{array}\right]$ is the transformation from the world system to the camera system. Let’s denote the *i*-th column of the rotation matrix R by $r_{i}$, the *i*-th column of the linear projection H by $h_{i}$. With the knowledge that $r_{1}$ and $r_{2}$ are orthonormal, two constraints on the internal parameter can be obtained:

(4) $$\{\begin{array}{c} h_{1}^{T}A^{-T}A^{-1}h_{2}=0\\ h_{1}^{T}A^{-T}A^{-1}h_{1}=h_{2}^{T}A^{-T}A^{-1}h_{2} \end{array}$$

Since one calibration pose can provide two constraints according to Equation (4), the internal parameter A can be calculated from at least three poses. Once A is known, the external parameter $\left\{R_{i},t_{i}|i=1..n\right\}$ for each calibration pose can be calculated according to Equation (5):

(5) $$\{\begin{array}{c} \begin{array}{l} r_{1}=\lambda A^{-1}h_{1}\\ r_{2}=\lambda A^{-1}h_{2}\\ r_{3}=r_{1}\times r_{2} \end{array}\\ t=\lambda A^{-1}h_{3} \end{array}\;\mathrm{with}\;\lambda =1/\left\|A^{-1}h_{1}\right\|$$

Under the linear projection model, for one camera pixel m, distortion will cause the deviation Δm between real coordinate and the reprojection $\hat{m}$:

(6) $$\Delta m=\hat{m}(A,R,t,M)-m$$

where M is the corresponding world coordinate of pixel m. Because distortion is a systematic error for a camera with fixed focal lens and short work distance, the deviation should be a fixed value for different calibration poses. By compensating the distortion with the average deviation $\bar{\Delta m}$ of n calibration poses according to Equation (7), a corrected coordinate $m^{*}$ accurately matching the linear model can be obtained:

(7) $$m^{*}=m+\bar{\Delta m}$$

Until now, the initial value of internal parameter A, external parameter $\left\{R_{i},t_{i}|i=1..n\right\}$, and distortion deviation $\bar{\Delta m}$ are obtained, and then they are recalculated and optimized through an iterative loop to minimize the following function with Levenberg-Marquardt Algorithm:

(8) $$\sum_{i=1}^{n}\sum_{j=1}^{k}\|m^{*}(m,\bar{\Delta m})-{\hat{m}(A,R_{i},t_{i},M_{ij})\|}^{2}$$

### 2.3. Compensation Algorithm for Phase Target Error

**Figure 4:**
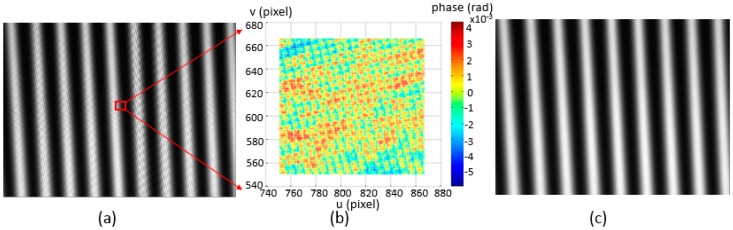
Error analysis of phase map. (**a**) Focused fringe pattern; (**b**) the difference between a phase map section and its fitting plane; (**c**) out-of-focus fringe pattern.

When the phase target is placed in the depth of focus of the camera, an observable deformation on the captured fringe pattern appears as shown in Figure 4a. The deformation comes from the recorded pixel grids on the LCD screen and also the moiré fringe created by the interference between camera pixels and LCD pixels. Taking a small area located at the middle of a calculated phase map, the influence can be seen from the difference between the phase value and its fitting plane, as shown in Figure 4b. In order to enhance the location accuracy of the phase target and guarantee an appropriate input for the following calibration process, a noise compensation algorithm is researched to refine the phase target. A Gaussian filter can be applied to the captured fringe patterns as the virtual defocusing technique used in [19]. However, it is difficult to determine the size of the filter. A flexible approach to eliminate the influence is to locate the phase target at an out-of-focus position of the camera by moving the target position or adjusting the camera focus, as shown in Figure 4c.

**Figure 5:**
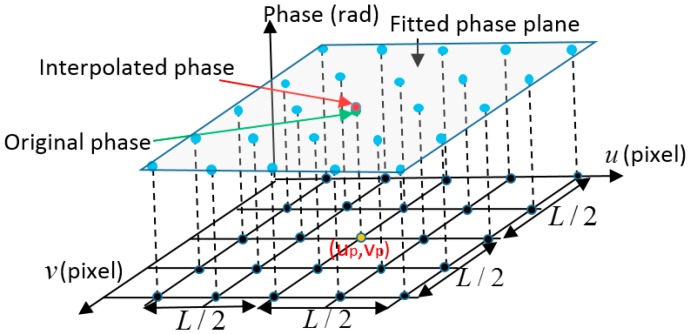
Principle of fitting and interpolation method.

Because the obtained absolute phase map is continuous and smooth, phase data can be smoothed by applying a fitting and interpolation method as shown in Figure 5. For a camera pixel $\left(u_{p},v_{p}\right)$ as the yellow dot shown in Figure 5, its original phase is represented as the green dot shown in Figure 5. Near the point on the phase surface, there is only a small difference between the phase surface and the tangent plane. Therefore a phase plane (such as the French grey plane shown in Figure 5) can be fitted with the phase values (the blue dots) of the neighboring *L*/2 pixels using the least square algorithm. Based on the fitted plane, an interpolated phase value (the red dot) can be calculated by smoothing the original phase according to the fitted plane based on cubic polynomial interpolation. Using a bigger fitting window (*L*) is more effective to remove errors and to enhance the accuracy of phase data. However based on Equations (1)–(3), the relationship between the obtained phase map and camera pixel coordinate (u,v) can be obtained by Equations (9) and (10), which are not linear equations, so that the captured phase map is a curve surface.

(9) $$\varphi_{x}=\frac{2\pi \cdot (h_{11}\cdot u+h_{12}\cdot v+h_{13})}{n_{p}\cdot p\cdot (h_{31}\cdot u+h_{32}\cdot v+h_{33})}$$

(10) $$\varphi_{y}=\frac{2\pi \cdot (h_{21}\cdot u+h_{22}\cdot v+h_{23})}{n_{p}\cdot p\cdot (h_{31}\cdot u+h_{32}\cdot v+h_{33})}$$

where $h_{ij}$ (i=1..3; j=1..3) is the elements of inverse matrix of H. Therefore, if the applied window is too big, the slope data in the window will have an obvious variation and the calculated point will be inaccurately adjusted.

**Figure 6:**
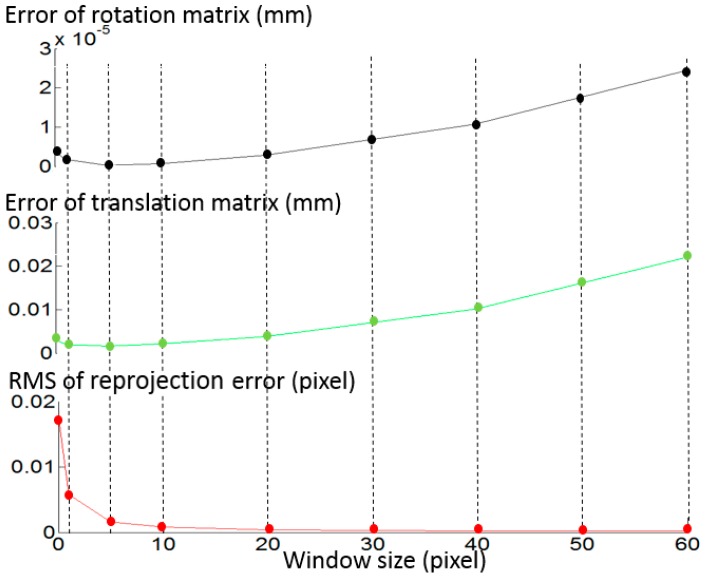
The variety of reprojection error, error of rotation matrix and error of translation matrix along with the variety of window size.

In order to optimize the fitting window size, a simulation experiment has been conducted as shown in Figure 6. Random phase error is the only error source in the simulation. Along with the window size increasing from 0 (no fitting and interpolation applied) to 5 pixels, the RMS (root mean square) of the reprojection error decreases dramatically (as shown by the red line). In this process, a reduction also happens in the error of rotation matrix *R* and the error of translation matrix *t* (as shown by the black and green lines, respectively). However, when the window size increases from 5 pixels to 60 pixels, though the RMS of the reprojection error decreases from 0.0016 pixels to 0.000172 pixels, the error of the rotation matrix and the translation matrix both increase. The simulation shows that five pixels is an appropriate fitting window size, which can reduce phase noise and not excessively adjust the original phase value.

## 3. Results and Discussion

### 3.1. Simulation Study

**Figure 7:**
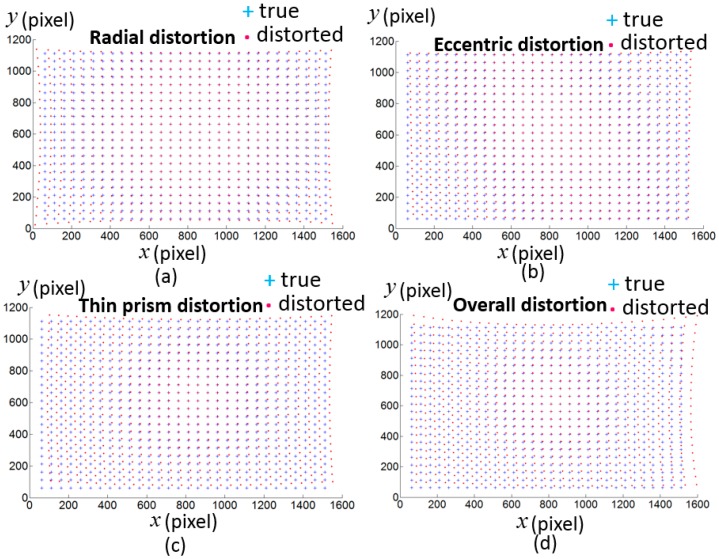
The simulated distortion on the pixel coordinate. (**a**) Radial distortion ; (**b**) eccentric distortion; (**c**) thin prism distortion; (**d**) the overall distortion caused by radial distortion, eccentric distortion and thin prism distortion.

**Figure 8:**
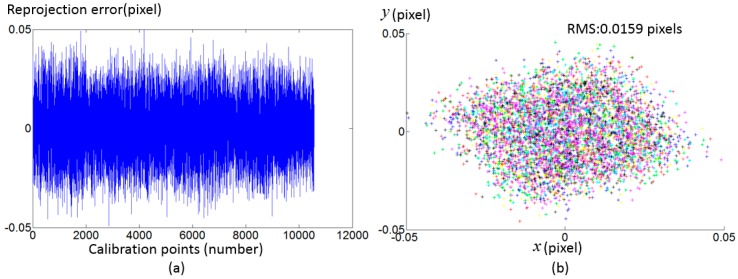
Calibration result with the simulated data. (**a**) Reprojection error of calibration points in terms of eight calibration poses; (**b**) Reprojection error in terms of pixel coordinate. Different calibration poses are distinguished with eight colors.

A simulation work has been done to test the proposed non-parameter-based distortion compensation method. The size of the simulated camera image is 1616 × 1216 with the principal point at (828,628) pixel. The scale factors along the u and v axes are 3543 pixel and 3522 pixel respectively. Radial distortion $\left(\Delta_{ur},\Delta_{vr}\right)$, eccentric distortion $\left(\Delta_{ue},\Delta_{ve}\right)$ and thin prism distortion $\left(\Delta_{up},\Delta_{vp}\right)$ are simulated according to Equation (11) with the coefficients $k_{1}=3\times 10^{-8}$ pixel, $k_{2}=3\times 10^{-14}$ pixel, $k_{3}=1\times 10^{-20}$ pixel, $k_{4}=1\times 10^{-26}$ pixel, $p_{1}=1\times 10^{-5}$ pixel, $p_{2}=1\times 10^{-5}$ pixel, $s_{1}=5\times 10^{-5}$ pixel, $s_{2}=5\times 10^{-5}$ pixel, and the distortion center is (808,608) pixel.

(11) $$\{\begin{array}{c} \begin{array}{l} \Delta_{ur}=u_{p}(k_{1}r^{2}+k_{2}r^{4}+k_{3}r^{6}+k_{4}r^{8})\\ \Delta_{vr}=v_{p}(k_{1}r^{2}+k_{2}r^{4}+k_{3}r^{6}+k_{4}r^{8}) \end{array}\\ \begin{array}{l} \Delta_{ue}=2p_{1}u_{p}v_{p}+p_{2}(u_{p}^{2}+3v_{p}^{2})\\ \Delta_{ve}=p_{1}(3u_{p}^{2}+v_{p}^{2})+2p_{2}u_{p}v_{p}\\ \Delta_{up}=s_{1}(u_{p}^{2}+v_{p}^{2})\\ \Delta_{vp}=s_{2}(u_{p}^{2}+v_{p}^{2}) \end{array} \end{array}\;\mathrm{with}\;r=\sqrt{u_{p}^{2}+v_{p}^{2}}$$

where $\left(u_{p},v_{p}\right)$ is the ideal pixel. Figure 7 shows the simulated ideal pixels and distorted pixels. 8 LCD pose are simulated with $r_{1}={\left[2.7695^{\circ },-2.7856^{\circ },-0.1811^{\circ }\right]}^{T}$, $t_{1}={\left[0.7916,-0.4575,-2.7113\right]}^{T}$, $r_{2}={\left[2.8293^{\circ },-2.7519^{\circ },-0.2241^{\circ }\right]}^{T}$, $t_{2}={\left[0.7275,-0.3875,2.7896\right]}^{T}$, $r_{3}={\left[3.0690^{\circ },-2.6283^{\circ },-0.2358^{\circ }\right]}^{T}$, $t_{3}={\left[0.5033,-0.4775,2.8014\right]}^{T}$, $r_{4}={\left[2.8311^{\circ },-2.7483^{\circ },-0.3103^{\circ }\right]}^{T}$, $t_{4}={\left[0.7353,-0.0266,3.1896\right]}^{T}$, $r_{5}={\left[2.8887^{\circ },-2.7270^{\circ },-0.0773^{\circ }\right]}^{T}$, $t_{5}={\left[0.7295,-0.4887,2.8933\right]}^{T}$, $r_{6}={\left[3.0078^{\circ },-2.6686^{\circ },-0.1458^{\circ }\right]}^{T}$, $t_{6}={\left[0.5983,-0.4071,2.9964\right]}^{T}$, $r_{7}={\left[-3.1212^{\circ },-2.5625^{\circ },-0.3108^{\circ }\right]}^{T}$, $t_{7}={\left[0.3537,-0.2911,3.1097\right]}^{T}$, $r_{8}={\left[2.8760^{\circ },-2.7257^{\circ },-0.2398^{\circ }\right]}^{T}$, $t_{8}={\left[0.7231,-0.0588,3.3075\right]}^{T}$. Random noise from 0 to 0.005 radian are added in the phase target. Figure 8 shows the reprojection error with the proposed calibration method. The result demonstrates the proposed calibration method can effectively compensate the camera distortion from complex distortion source.

### 3.2. Experiment Study

**Figure 9:**
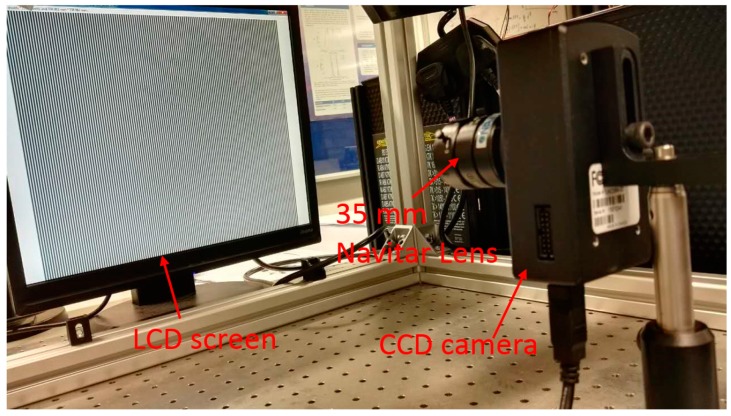
The experimental setup.

**Figure 10:**
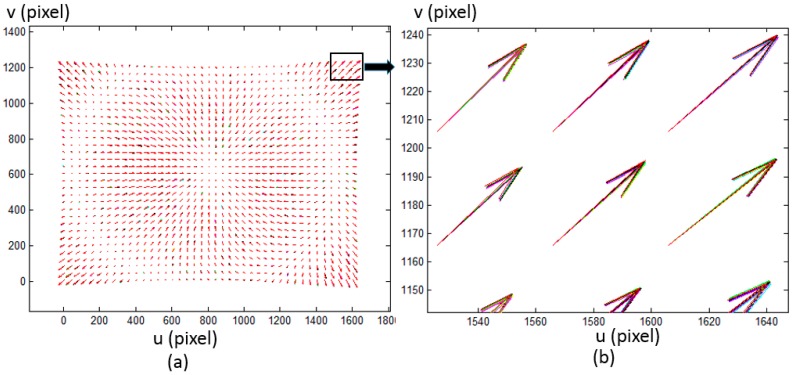
Distortions of the camera pixels in terms of 11 calibration poses, which are distinguished with 11 colors. For the purpose of clear illustration in the figures, only part of the pixels are shown and the vector scales are different. (**b**) is the magnification of (**a**).

**Figure 11:**
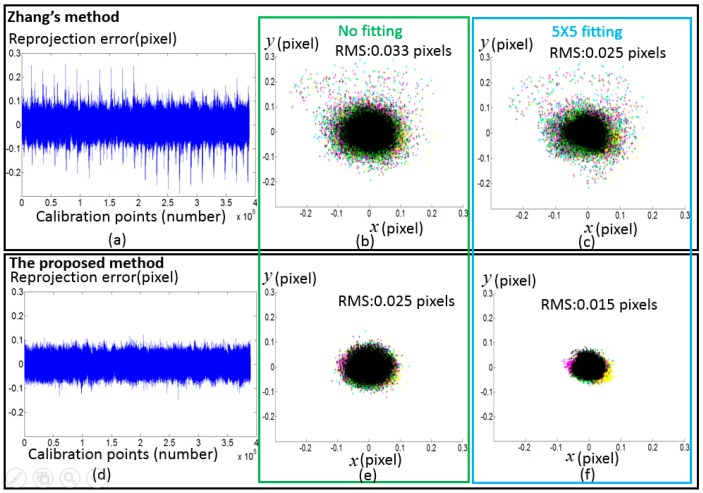
Calibration results with the two calibration approach. (**a**) Reprojection error of calibration points with the conventional method before using the fitting method; (**b**) Reprojection error in terms of pixel coordinate with the conventional method before using the fitting method; (**c**) Reprojection error in terms of pixel coordinates with the conventional method after using the fitting method; (**d**) Reprojection error of calibration points with the proposed method before using the fitting method; (**e**) Reprojection error in terms of pixel coordinates with the proposed method before using the fitting method; (**f**) Reprojection error in terms of pixel coordinates with the proposed method after using the fitting method; Different calibration poses are distinguished with 11 colors.

To verify the proposed method, calibration experiments have been conducted on a Lumenera CCD camera (Model Lw235M). The camera’s CCD sensor has a resolution of 1616 × 1216. The camera lens is a Navitar lens with a 35 mm fixed focal length. A LCD monitor (Dell E151Fpp with a resolution of 1024 × 768, Philips, Shanghai, China) was used as the phase target. The pixel pitch of the LCD is 0.297 mm. The setup of the hardware system is illustrated in Figure 9. During the calibration process, the phase target was placed at 11 different poses. Absolute phase maps were obtained by using an eight-step phase shifting algorithm and the optimum frequency selection method [19,20], and thereafter they were smoothed by fitting and interpolating algorithm with a 5 × 5 fitting window. Every 10th camera pixel was selected to form a grid with the size of 161 × 121 as the input to calibrate the cameras with the corresponding points in the world coordinates. The distortions of the selected pixels under the linear projection model are shown in Figure 10. Since Zhang’s calibration approach [1] and the calibration toolbox [10] are very popular in practical applications, a comparative experiment has been made between the proposed method and Zhang’s approach as shown in Figure 11. Zhang’s method can be classified as four steps: (1) To discover the corresponding position between feature points and camera pixels; (2) To calculate the initial values of a camera’s parameter and external parameters based on the pinhole model; (3) To consider distortion compensation based on a mathematical model; (4) To optimize the initial camera parameters, external parameter and distortion parameter with the Levenberg–Marquardt algorithm. The feature points can be detected from a chessboard as used in Zhang’s article [1] or another calibration target. To avoid the difference caused by the extraction accuracy of different calibration target, the same camera pixels and the corresponding physical points were used to test the two methods. Using the proposed method, the RMS of the reprojection error is 0.025 pixels before applying the fitting method and 0.015 pixels after applying the fitting method respectively. In contrast, the RMS of the reprojection error of the conventional method is 0.033 pixels before applying the fitting method and 0.025 pixels after applying the fitting method respectively, which are 1.3 and 1.6 times respectively worse than the proposed method.

## 4. Conclusions

A novel camera calibration method with an iterative distortion compensation algorithm based on a full-field camera pixel and dense phase target has been proposed. To enhance the location accuracy of the phase value, techniques of defocus, fitting and interpolation are studied. Without relying on the distortion mathematical model, the proposed calibration method can obtain pixel-by-pixel distortions and high calibration accuracy. Next steps will include the proposed calibration method applied in a deflectometry system.

It would have been preferable if the proposed method were compared with other non-parameter-based distortion compensation approaches. However, there were several different factors to consider in this experiment: (1) It is difficult to obtain the open code for these approaches; (2) Some of these approaches are based on a particular calibration target, therefore it is impossible to guarantee the comparative experiment having the same calibration input. In fact, the simulation study in this paper can demonstrate the superiority of the proposed method, as the other non-parameter-based approaches cannot handle the eccentric distortion and the thin prism distortion [16,17], or cannot obtain the internal parameter of the camera [18]. This simulation study shows that the proposed method can effectively calibrate a camera with a complex distortion condition. Based on this point, the conclusion can be made that the proposed method is superior to the other non-parameter-based distortion compensation approaches.
